# Influence of Bacteria of the Genus *Pseudomonas* on Leguminous Plants and Their Joint Application for Bioremediation of Oil Contaminated Soils

**DOI:** 10.3390/plants11233396

**Published:** 2022-12-06

**Authors:** Elena Kuzina, Svetlana Mukhamatdyarova, Yuliyana Sharipova, Ainur Makhmutov, Larisa Belan, Tatyana Korshunova

**Affiliations:** 1 Ufa Institute of Biology, Ufa Federal Research Centre, Russian Academy of Sciences, 450054 Ufa, Russia; 2 Department of Environmental Protection and Prudent Exploitation of Natural Resources, Ufa State Petroleum Technological University, 450044 Ufa, Russia

**Keywords:** *Pseudomonas*, leguminous plants, growth stimulation, nodulation, bioremediation of oil-contaminated soil

## Abstract

The modern approach to the creation of biological products to stimulate plant growth is based on the study of specific inter-bacterial interactions. This study describes the impact that the introduction of strains of the genus *Pseudomonas* has on annual and perennial leguminous plants and the ecosystem of the leguminous plant—the indigenous microbial community. The objects of research under the conditions of vegetation experiments were plants of field peas (*Pisum sativum* L.), white lupine (*Lupinus albus* L.), chickpea (*Cicer arietinum* L.), alfalfa (*Medicago sativa* subsp. *varia* (Martyn) Arcang.), and white sweet clover (*Melilotus albus* Medik.). For the treatment of plant seeds, a liquid culture of strains of growth-stimulating bacteria *Pseudomonas koreensis* IB-4, and *P. laurentiana* ANT 17 was used. The positive effect of the studied strains on the germination, growth and development of plants was established. There was no inhibitory effect of inoculants on rhizobia; on the contrary, an increase in nodule formation was observed. The possibility of recultivation of oil-contaminated soil using chickpea and alfalfa as phytomeliorants and growth-stimulating strains *P. koreensis* IB-4, *P. laurentiana* ANT 17 as inoculants was evaluated. It is proved that seed treatment improved the morphological parameters of plants, as well as the efficiency of oil destruction.

## 1. Introduction

To date, a number of mechanisms of direct and indirect positive effects of bacteria on plants are known [1,2,3]. In most cases, the same plant growth-promoting bacteria (PGPB) can be endowed with properties of various natures that are useful for plants [4,5]. However, the issue of the effect of introduced microorganisms on the native soil microbiota and naturally formed microbial-plant associations remains poorly understood.

It has been established that the effectiveness of a biological preparation depends not only on its multifunctionality, but also on how successfully biological control agents manage to occupy an ecological niche in an already existing community [6]. The symbiosis of leguminous plants and rhizobia is one of the best known examples of a balanced phytomicrobiome. Nodule bacteria contribute to an increase in the yield of legumes, providing the latter with additional nitrogen, but such properties of PGP microorganisms as the synthesis of phytohormones and biocontrol of phytopathogens are not often found in them [7]. Therefore, it may be of interest to study the possibilities of increasing the potential of leguminous plants through the introduction of growth-stimulating microorganisms, for example, bacteria of the genus *Pseudomonas*. Representatives of this genus increase the availability of mineral nutrition elements for plants [8,9,10]. They produce growth-regulating metabolites [11,12,13] and induce resistance to abiotic and biotic stresses [14,15,16]. *Pseudomonas* can affect plants by suppressing the development of phytopathogenic organisms [5,17], as well as by reducing the content of harmful chemical compounds and heavy metals in the soil [15,18]. Pseudomonas are able to successfully colonize the plant rhizosphere and survive in it [19,20]. The strains of *Pseudomonas koreensis* IB-4 and *P. laurentiana* ANT 17 that we used in this study belong to PGPB, although they differ somewhat in their properties. Previously, it was shown that IB-4 is capable of nitrogen fixation and that it synthesizes substances of a cytokinin nature, while ANT 17 synthesizes indolyl-3-acetic acid (IAA). Both cultures are antagonists of phytopathogenic micromycetes [21,22].

Hydrocarbons are recognized as the main environmental pollutants [23]. Bioremediation is an effective option for restoring oil-polluted ecosystems using the degradation capabilities of plants and microorganisms. Representatives of the family *Fabaceae* are often used as phytomeliorants [24,25], since, compared to plants of other families, legumes provide not only the recultivation of polluted soil, but also help restore the balance of nitrogen and carbon [26]. Most often, various types of alfalfa and clover and their legume-cereal crop mixtures are used to clean the soil from oil [27,28].

It is obvious that the morphological changes that occur with plants under the influence of oil pollution are due, among other things, to difficulties with the supply of moisture to the roots. Therefore, drought-resistant legumes that can tolerate a lack of moisture in the soil for a long time are of particular interest. Chickpea (*Cicer arietinum* L.) is one of the best known xerophytic leguminous crops [29,30]. In our opinion, its potential as a phytomeliorant has not been sufficiently studied, and there are only a few publications devoted to this issue [31,32].

The purpose of this study was to evaluate the impact of the introduction of PGP bacteria of the genus *Pseudomonas* on the leguminous plant–indigenous microbial community ecosystem using various legumes as an example. This would make it possible to further use the results obtained in the creation of biological preparations to stimulate the growth and increase the yield of these valuable agricultural crops. In addition, the work included the study of the response of leguminous plants to the presence of oil and treatment with growth-stimulating pseudomonads, as well as the study of the effectiveness of the use of associations of legumes and *Pseudomonas* bacteria to reduce the content of hydrocarbons in the soil, which is important for the development of environmentally friendly approaches to the cleanup and restoration of anthropogenically disturbed territories. It was assumed that the PGPB strains *P. koreensis* IB-4 and *P. laurentiana* ANT 17 would have a positive effect on the growth and development of legumes, including under conditions of oil pollution, and would enhance the biodegradation of the pollutant in the soil. It should be emphasized that the species *P. koreensis* is fairly well described in the literature, including as a decomposer of petroleum hydrocarbons [33,34], while the species *P. laurentiana* was discovered relatively recently [35]. In this regard, studies on its ability to utilize oil are still rare [36].

## 2. Results

### 2.1. The Effect of Bacteria on Plants

**Figure 1:**
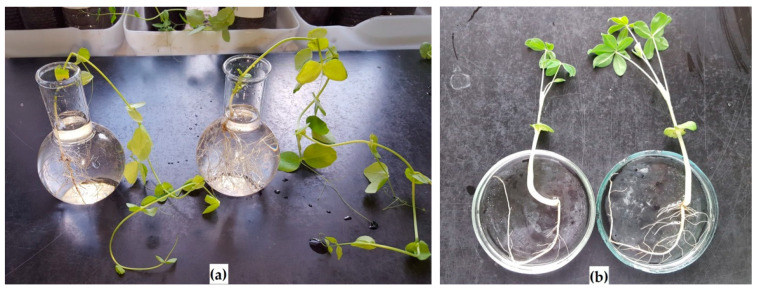
Influence of the introduction of bacterial strains on the development of leguminous plants: (**a**) Peas, left—control, right—IB-4 strain; (**b**): Lupine, left—control, right—ANT 17 strain.

Strains IB-4 and ANT 17 had a positive effect on the germination of seeds of leguminous plants (Table 1), especially alfalfa. The seeds of this culture had the lowest germination, which increased by 10–15% after bacterization. Both strains stimulated the development of only pea shoots and only the underground part of chickpea (Table 1), despite the fact that ANT 17 synthesizes IAA [22], which promotes the growth (elongation and branching) of roots [37], and IB-4 produces cytokinins [21], activating to a greater extent the growth of shoots than of roots [3]. In general, the introduced strains were more active on peas, lupine and chickpea (Figure 1). The positive effect was less noticeable or absent on alfalfa and sweet clover.

The formation of the first nodules was observed approximately on the 10th day after the emergence of shoots, but nodules were not detected on the roots of chickpea throughout the experiment. Inoculation with bacteria generally had a positive effect on the process of nodulation, but plants of various genera responded differently to the introduction of PGPB (Table 1). On day 21 the number of nodules of peas treated with the ANT 17 strain exceeded the control values by almost six times (Table 1); at alfalfa inoculated with this microorganism, by two times. On the 42nd day, in all variants of the experiment, where the plants were inoculated with the ANT 17 strain, the number of nodules of the plants was more than in the control version (1.3–2.3 times). For the IB-4 strain on the 42nd day, an increase in the formation of nodules was noted only on peas and alfalfa (by 2.3 and 1.5 times, respectively).

### 2.2. Nitrogen Content in Soil and Plants

**Figure 2:**
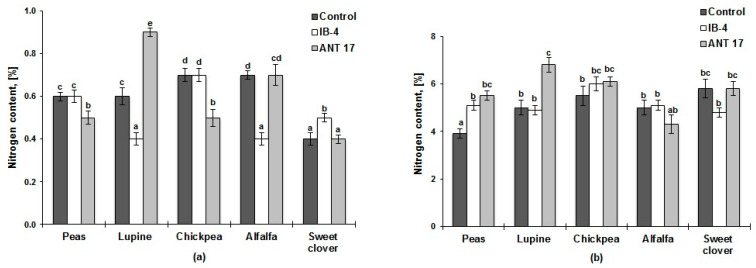
Nitrogen content: (**a**) In the soil after growing plants of peas, white lupine, chickpea, alfalfa changeable, white sweet clover; (**b**) In plants. IB-4 and ANT 17 are variants of the experiment with the introduction of *P. koreensis* IB–4 and *P. laurentiana* ANT 17, respectively. Statistically different means values are marked with different letters (*p* ≤ 0.05).

After harvesting the plants, compared to the control, the nitrogen content in the soil increased only in the vegetation vessels where lupine had been inoculated with the ANT 17 strain (initial content—0.5%) (Figure 2a). Compared to the control, increased nitrogen accumulation of plants was noted in variants where bacterization increased the number of nodules significantly (both strains on peas, the ANT 17 strain on lupine) (Table 1 and Figure 2b).

### 2.3. The Number of Microorganisms in the Rhizosphere of Plants

The maximum number of heterotrophic microorganisms in large-seeded plants (peas, chickpea, lupine) mainly fell in the initial period of development. In fodder grasses, on the contrary, an increase of the number of this group of microorganisms occurred by the end of the experiment (Table 2). Bacterization had an impact on the ammonifying microflora. On the 42nd day of the experiment (the beginning of the flowering phase), a slight decrease of the number of ammonifiers in the rhizosphere of peas, lupine and chickpea inoculated with bacterial strains was observed. Bacterization had no effect on the number of nitrogen-fixing microorganisms. In the middle of the experiment, the largest number of representatives of this group was found in the chickpea rhizosphere, but at the end of the experiment, their number in chickpea variants did not differ from the data obtained for other plants. At the end of the experiment, there was a tendency to increase the number of nitrogen-fixing microorganisms in the rhizosphere of alfalfa and sweet clover. Bacterization contributed to the suppression of the development of microscopic fungi in the rhizosphere of plants in the first half of the experiment. By the end of the experiment, the difference in the number of micromycetes in the control and experimental variants decreased or turned out to be statistically unreliable.

### 2.4. The Effect of Bacteria on Plants in Conditions of Pollution

**Figure 3:**
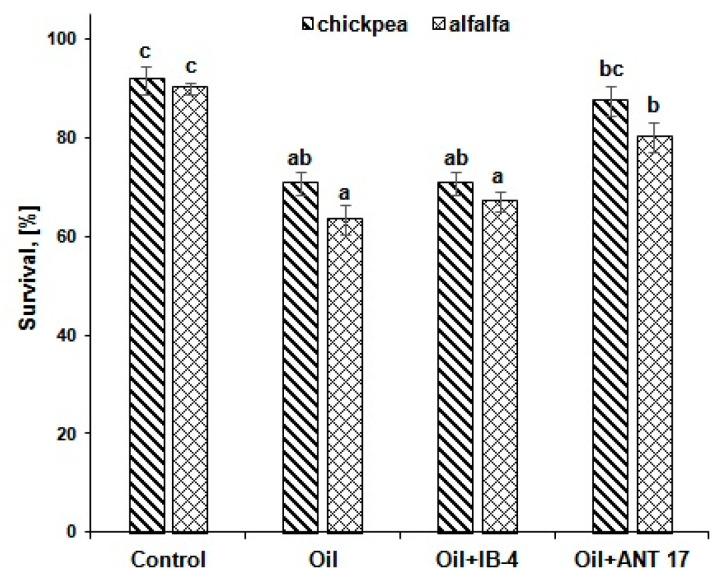
The influence of bacterial introduction on plant survival under conditions of oil pollution. IB-4 and ANT 17 are variants of experiments with the introduction of *P. koreensis* IB-4 and *P. laurentiana* ANT 17, respectively. Statistically different means values are marked with different letters.

The second stage of research devoted to bioremediation began with an assessment of the response of chickpea and alfalfa to the presence of oil in the soil. It was shown that the survival rate of both plants on oil-contaminated soil decreased: in chickpea, by 21% and in alfalfa, by 27% (Figure 3). At the same time, against the background of oil pollution, bacterization of seeds with the ANT 17 strain increased the survival rate of chickpea and alfalfa by 17%.

**Figure 4:**
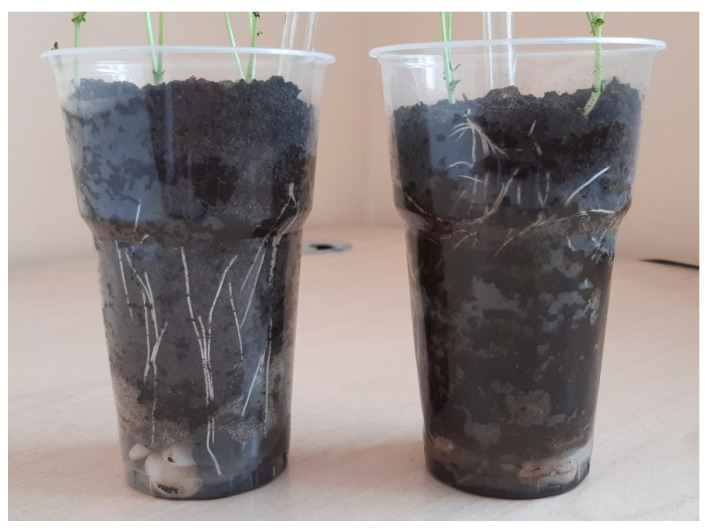
The root system of chickpea plants. **Left**—control, **right**—oil-contaminated soil.

At the early stages of the experiment, the main mass of chickpea roots in the vessels with oil was located in the upper soil layer, i.e., the plant experienced significant stress from the presence of the pollutant (Figure 4). At the same time, there were no statistically significant differences between the dry weight of chickpea roots and of chickpea shoots grown in the control variant and in the soil containing oil (Table 3). The introduction of bacteria stimulated plant growth in the presence of a pollutant. In inoculated chickpea plants, the dry weight of the shoot increased by 10.1–18.0% compared to the variant containing oil, but without the introduction of microorganisms, and by 14.7–23.0% compared to the control. Treatment with the ANT 17 strain also significantly accelerated the growth of chickpea roots in oil-contaminated soil, where the control indicators were exceeded by 35.4%.

The toxic effect of oil on alfalfa plants was expressed, first of all, in a significant slowdown in the growth of shoots. By the end of the experiment, alfalfa plants growing on polluted soil had four true leaves, while on clean soil they had an average of 5–6 leaves. The dry weight of the above-ground mass in the variant with oil was four times less compared to the control. Bacterization helped plants cope with stress. When using the IB-4 strain, the weight of the shoot increased by 69.7% compared with untreated plants in oil-containing soil. Outwardly, this effect was expressed not in an increase in the number of leaves, but in an increase in the size of leaf blades. In contrast to the shoots, the presence of the pollutant did not have an inhibitory effect on the root system of alfalfa.

Thus, it was found that in the soil with oil (including in the presence of PGPB), chickpea showed a tendency to a slight increase in the growth of aboveground and underground parts of plants; therefore, the presence of the pollutant had almost no effect on the ratio of root mass to shoot mass (Table 3). In alfalfa, this indicator in the soil with oil increased to 0.74 against 0.12 in the control variant. Bacterization had a more noticeable effect on the growth of shoots than roots and led to a decrease in the ratio root/shoot from 0.74 to 0.51–0.55.

The formation of the first nodules in alfalfa on polluted soil occurred on the 17th day of the experiment. The introduction of oil led to a decrease in nodulation by 2.4 times (Table 3). Inoculation with bacterial strains increased the number of nodules, but they were still less than in the control.

### 2.5. The Number of Microorganisms in the Rhizosphere of Plants in Conditions of Pollution

Oil pollution caused an increase in the number of petroleum-degrading microorganisms in the rhizosphere of alfalfa and chickpea by 3.7–4.7 times compared to the control (Table 4). The number of heterotrophic microorganisms in the rhizosphere of non-inoculated plants on contaminated soil increased by 2.2–2.5 times compared with the control, and in the case of inoculation, by 4.5–6.0 and 4.2–5.9 times in the variants with chickpea and alfalfa, respectively. The presence of the pollutant negatively affected the number of oligonitrophilic microorganisms, which decreased by one order. Oil pollution also led to a decrease in the number of micromycetes, but in the variants where chickpea and alfalfa seeds were inoculated with bacterial strains, this indicator remained at the control level.

### 2.6. Biodegradation of Hydrocarbons

**Figure 5:**
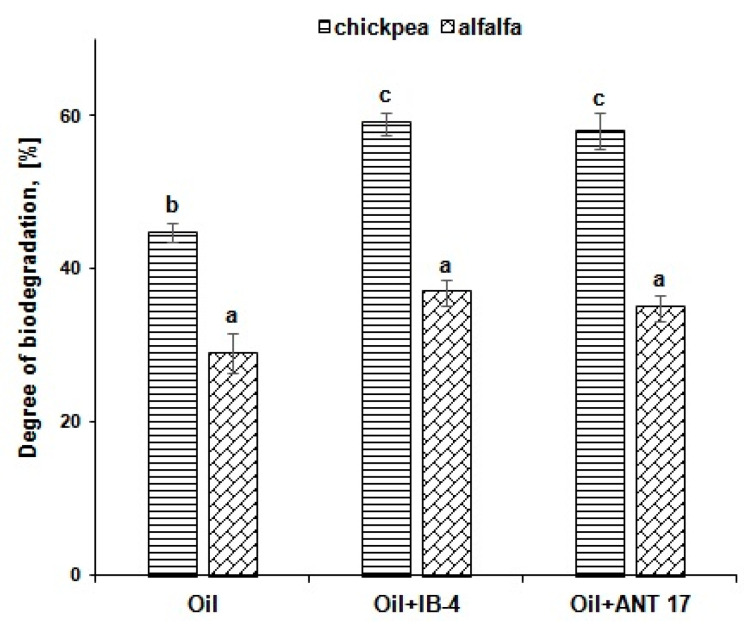
The degree of hydrocarbons biodegradation in various reclamation options. IB-4 and ANT 17 are variants of experiments with the introduction of *P. koreensis* IB-4 and *P. laurentiana* ANT 17, respectively. Statistically different means values are marked with different letters.

The degree of oil biodegradation in the soil with the use of chickpea was 45% for four weeks of the experiment, and 29% in the variant with alfalfa (Figure 5). Inoculation of chickpea plants with Pseudomonas strains increased this index by 13–14%. In the case of alfalfa, the introduction of bacteria did not have a noticeable effect on the process of oil decomposition.

## 3. Discussion

It is known that the introduction of PGPB does not always have a stimulating effect on the plant [38]. The effectiveness of bacterial treatments may vary even on different varieties of the same type of agricultural crop [39,40]. Legumes are very selective with respect to introduced bacterial strains and respond differently to the use of biological preparations [41,42]. In our experiment, the introduced strains were less effective on alfalfa and sweet clover. It is possible that the seed shell of these plants contains substances to the action of which the studied strains were not resistant [43].

The legumes used in this study differed significantly in the amount of acreage that is allocated for them. Obviously, if *Rhizobium leguminosarum* strains (peas microsymbiont) are distributed almost everywhere, then in the case of chickpea in soil where these plants have not been grown before (most European countries), suitable microsymbionts may be absent. Since rhizobium preparations were not used in this study, chickpea turned out to be the only crop on which nodules were not formed.

It is known that the phytohormones cytokinin and auxin are nodulating agents (stimulate the formation of nodules) [44]; however, for example, a high concentration of auxins can inhibit the formation of nodules [45]. Analysis of the nodulation shows that the studied strains did not show antagonism against an indigenous rhizobial bacteria; on the contrary, their ability to synthesize phytohormones [21,22] could lead to the increase in the number of nodules on plants.

Free-living and associative nitrogen-fixing microorganisms, along with rhizobia, contribute to the enrichment of the soil with atmospheric nitrogen. At the same time, part of the atmospheric nitrogen fixed by diazotrophs may remain inaccessible to plants, since it is localized in the soil as part of microbial biomass [46]. In our experiment, chickpea did not form nodules, but the amount of nitrogen absorbed by this plant turned out to be the same as in other legumes participating in the experiment, and the content of soil nitrogen increased markedly by the end of the experiment (Figure 2). Consequently, free-living and associative nitrogen-fixing microorganisms present in the soil, even in the absence of chickpea-specific nodule bacteria, provided nitrogen nutrition for this culture.

Microbiological analysis of the rhizosphere of legumes has shown that there are differences in the dynamics of the number of microorganisms in various representatives of this family (Table 2). This phenomenon is explained by differences in the life cycles and strategies for the accumulation of nutrients in annual and perennial legumes. At the end of the experiment, leguminous plants (peas, chickpea, lupine) had a phase of completion of active growth, which was associated with the outflow of nitrogen and other nutrients from the vegetative organs to the reproductive ones. At the same time, perennial crops (alfalfa, sweet clover) continued to actively develop and store nutrients in the root system, providing nutrition to the microbial community.

When such an important criterion as the survival of plants on soils contaminated with petroleum products is analyzed, it is believed that medium and high resistance is demonstrated by crops that retain more than 80% of plants from the number of seedlings [47]. In our experiment, with oil pollution (50 g/kg of soil), only 63% of alfalfa plants and 71% of chickpea plants survived for a month, but pre-sowing treatment with the ANT 17 strain increased this indicator to 80 and 88%, respectively (Figure 3). This fact confirms the advantage of using plant-microbial associations in reclamation activities compared to phyto- and microbiological remediation.

There is no consensus on which characteristics of seeds (size, weight, biological characteristics) increase the tolerance of plants to soil contamination. At the beginning of the experiment, we observed the relative resistance of chickpea to the toxic effects of oil. The observed effect may be due to the fact that the weight of its seeds exceeds the weight of alfalfa seeds by more than 150 times. Large cotyledons, acting as a receptacle of spare substances, allow the plant to adapt better to stressful environmental conditions at the initial stage of development. Indeed, at the beginning of the experiment, soil contamination with hydrocarbons did not affect the growth rate of the green mass of chickpea plants. Later, however, the leaves began to lose their green color and dry out, starting from the tips. In contrast, the toxic effect of oil on alfalfa culture was already noted at the stage of seedling emergence. At the same time, throughout the experiment, we did not observe yellowing and drying of leaves, although many authors indicate that chlorosis is a characteristic stress reaction of alfalfa to soil contamination with hydrocarbons [48].

At the beginning of plant growth, the root system usually develops faster than the aboveground part. In our experience, in clean soil, chickpea roots reached the bottom of the vessel on the third day. In the soil containing oil, the depth of root penetration was two times less (Figure 4). In addition, in the variant with contamination, a change in the direction of root growth was revealed, and they appeared above the soil surface. It is known that the negative impact of oil pollution on plants is not only due to the toxicity of its components; it also occurs indirectly through the influence on the physico-chemical properties of the soil [49,50,51]. The root system of plants is forced to adapt to existing conditions, using various mechanisms for this: changing the orientation of the roots relative to the substrate surface [52], hydrotropism and aerotropism [53].

Inhibition of shoot growth against the background of stress from the presence of oil in the soil is necessary for plants to reduce water evaporation and at the same time free up resources to support root growth and increase their absorption capacity. In our experiment, alfalfa plants on soil with oil showed a delay in the growth of stems, a decrease in the area of leaves and their number and, as a result, a significant decrease in the green mass (Table 3). In comparison with alfalfa, chickpeas did not show statistically significant differences between the data on dry biomass formed during four weeks on polluted and clean soils. Previously, we have already obtained similar results by comparing the resistance of leguminous plants to oil pollution. It has been shown that at the initial stages of development, small-seeded legumes are more vulnerable than plants with large seeds [54].

One of the main objectives of the study was to confirm the possibility of increasing the efficiency of phytoremediation by introducing bacteria. The joint use of plants and bacteria for the recultivation of soils contaminated with hydrocarbons is based on the mutually beneficial relationship that exists between them: the plant provides bacteria with easily accessible food sources, while the bacteria help plants survive in a toxic environment, stimulating their growth and reducing the concentration of harmful substances in the soil [39,55,56]. Good results have been obtained using biopreparations based on *Pseudomonas* strains together with plants of the legume family in the restoration of oil-contaminated lands [57,58,59,60]. However, associations of plants and PGPB are not always effective. According to Gilan et al. [61], the degree of soil purification with alfalfa decreased after its bacterization with *P. putida*. It is known that plants are selective with respect to microorganisms that form the microbiome of their rhizosphere [62]. PGPBs form biofilms on the roots of the host plant and secrete exopolysaccharides that interact with plant cell lectins, which can determine the specificity of the symbiosis; the effectiveness of symbiosis depends on the genotype of the bacterium and the genotype of the host plant [63]. The obtained results are consistent with the already mentioned studies on the effectiveness of the use of pseudomonas and legumes for cleaning oil-contaminated soils. It was found that seed treatment with strains IB-4 and ANT 17 reduced the negative effect of oil on the formation of green mass of plants (Table 3). Probably, an increase in plant productivity increased the intensity of exudate release. This, in turn, stimulated the activity of the hydrocarbon-oxidizing microbiota and accelerated the remediation process. At the same time, a more noticeable reaction of chickpeas to bacterization was found in oil-contaminated soil. The root system of chickpea was more developed than that of alfalfa (Table 3). Rapidly growing root systems produce more exudates [64], which leads to more active colonization of the rhizosphere and a more pronounced positive effect of bacteria on plants.

In a number of papers [62,65,66], the authors note the important role that various (exogenous) compounds, including pollutants, play in the formation of the composition of microbial communities of the rhizosphere. On the one hand, they can be toxic by suppressing certain soil microorganisms; on the other hand, pollutants can serve as a source of nutrition for community members. The data obtained by us on the number of nitrogen-fixing and hydrocarbon-oxidizing microorganisms in the soil containing oil can serve as a visual illustration of this statement (Table 4).

## 4. Materials and Methods

### 4.1. Plant Growth Conditions and Treatments

In the present work, we used seeds of the following plants of the legume family: field peas (*Pisum sativum* L.), variety Chishminsky 95; white lupine (*Lupinus albus* L.), variety Dega; chickpea (*Cicer arietinum* L.), variety Zavolzhsky; alfalfa (*Medicago sativa* subsp. *varia* (Martyn) Arcang.), variety Galia; and white sweet clover (*Melilotus albus* Medik.), variety Chermasan. Seeds were immersed in water to swell for one day and then placed on moistened filter paper for germination at room temperature. Germinated seeds were inoculated with microorganisms immediately before sowing at the rate of 10^5^ CFU/seed for large seeds (pea, lupin, chickpea) and 10^3^ CFU/seed for small seeds (alfalfa, sweet clover). Uninoculated seeds served as control.

In experiments to study the effect of growth-stimulating bacteria on the leguminous plant ecosystem-native microbial community, we used clay-illuvial chernozem (total humus 4.2%, total nitrogen 0.5%, mobile phosphorus 5.6 mg/100 g of soil; pH of the aqueous extract 6.3). Drainage and soil (1300 g) were placed in the vegetation vessels. Germinated seeds (pea, lupine, chickpea—5 pieces each, alfalfa and sweet clover—10 pieces each) were planted to a depth of 1–3 cm. The experiment was carried out for 42 days at room temperature and natural light. Humidity throughout the experiment was maintained at 60% of the total moisture capacity. Plant and soil samples were taken on days 21 and 42. The plants were removed together with the soil monolith, the root system was carefully washed, and then the morphological parameters (shoot length, root length, number of leaves) and the number of formed nodules were evaluated. In dried plants and in the soil, the content of total nitrogen was determined by the Kjeldahl method [67].

In the experiment on bioremediation of oil-contaminated soil, chickpeas and alfalfa were used as phytomeliorants. Soil (see above) was placed in the vessels, preliminarily mixed with sand (soil:sand ratio 9:1) and contaminated with commercial oil of the Urals brand at a concentration of 50 g/kg of soil by weight. Plants grown on uncontaminated soil served as control. Humidity at the beginning of the experiment was maintained at 80%, and then at 60% of the total soil moisture capacity. Plant exposure lasted for 28 days. At the end of the experiment, the number of surviving plants, nodulation, and the air-dry mass of shoots and roots were evaluated. Plant survival was calculated as the ratio of the number of plants remaining at the end of the experiment to the number of seedlings planted (%). The efficiency of bioremediation of oil-contaminated soil was assessed by the degree of biodegradation of hydrocarbons in the soil.

### 4.2. Cultivation of the Microorganisms and Analysis of Their Number

To study the effect of growth-stimulating bacteria on plant growth, including under oil pollution, we used bacterial strains of *Pseudomonas koreensis* IB-4 (VKM B-2830D) and *P. laurentiana* ANT 17 that are kept in the collection of the Ufa Institute of Biology. Previously, the authors isolated these microorganisms from arable soil (Russia, Republic of Bashkortostan, Mechetlinskii district) (*P. koreensis* IB-4), and from activated sludge biological treatment facilities of an oil refinery (Russia, Orenburg region, city of Orsk) (*P. laurentiana* ANT 17) [22,23]. Bacteria were grown on King B liquid nutrient medium (g/L) [68] for 72 h at a temperature of 28 °C. Aeration of the medium was provided by rotating flasks (160 rpm) in an orbital shaker-incubator ES-20/60 (SIA BIOSAN, Riga, Latvia). The number of cells in the culture was measured by applying serial dilutions to the nutrient agar (g/L): peptone—10.0, yeast extract—3.0, NaCl—5.0, glucose—1.0, agar-agar—15.0, and then counting the number of CFU.

In order to estimate the microbial counts in rhizosphere soil, a serial dilution of soil suspension was used. The number of heterotrophic microorganisms was measured by application to the nutrient agar (see above). For measuring the number of petroleum-degrading bacteria, we used Raymond agar [69], supplemented with 0.1 g of sterile diesel fuel as the only source of carbon, smeared on the agar surface of each plate. To measure the number of nitrogen-fixing and oligonitrophilic microorganisms, we used Ashby medium, and the number of micromycetes was measured on the Czapek-Dox agar [70]. The incubation period at 28 °C was three days on nutrient agar, and five days on the Raymond agar, the Ashby agar, and the Czapek-Dox agar. The average number of colonies was calculated in ten agar plates.

### 4.3. Analysis of the Content of Hydrocarbons in the Soil

Total petroleum hydrocarbons in the soil samples were measured using the EPA 3540C method. Then, 10 g of soil samples were packed in filter paper and extracted in a Soxhlet extractor with 300 mL of hexane for 8 h at six extraction cycles per hour. The extraction product was transferred to a glass column filled with glass wool and Na_2_SO_4_ to remove any water it contained. The extract was collected in a flask for subsequent evaporation of the solvent using a rotary evaporator Rotavapor R-100 (Buchi Labortechnik AG, Flawil, Switzerland) until a final volume of 2 mL was reached. The concentrated solution was poured into a pre-weighed glass beaker and dried until a constant weight was reached. The total petroleum hydrocarbons present in the samples were then quantified by gravimetric analysis with a weighing accuracy of up to 0.1 mg.

The degree of hydrocarbons biodegradation (*D*) was calculated from the formula:

(1) $$D\;\left(\%\right)=\frac{C_{0}-C_{1}}{C_{0}}\cdot 100,$$

where *C*_0_ is the initial concentration of oil hydrocarbons (50 g/kg soil), and *C*_1_ is the concentration of oil hydrocarbons in the samples at the end of the experiment.

### 4.4. Statistical Analysis

The data were processed using Statistica (Statsoft) software (version 10). In figures and tables, data are presented as mean ± standard error (SE). The significance of differences was assessed by ANOVA followed by Duncan’s test (*p* ≤ 0.05).

## 5. Conclusions

The present study was devoted to the study of the reactions of leguminous plants to PGPB of the genus *Pseudomonas*, and it also studied the potential of chickpea and alfalfa as phytomeliorants for the restoration of oil-contaminated soils in combination with bacterial treatments.

Different types of legumes (field peas, chickpeas, white lupine, alfalfa, white sweet clover) reacted differently to the introduction of growth-stimulating bacteria of the genus *Pseudomonas*. In general, inoculation of seeds with strains *P. koreensis* IB-4 and *P. laurentiana* ANT 17 increased germination, enhanced the growth of shoots and roots, improved nitrogen nutrition of plants, and stimulated the formation of nodules. An experiment on the restoration of oil-contaminated soil with chickpea and alfalfa plants showed that the experimental crops showed an average resistance to oil (50 g/kg of soil). The toxic effect of the pollutant was expressed in a decrease in the survival of both legumes. However, they differed in morphological changes that occurred to them under the influence of oil. Bacterization of seeds with PGPB strains *P. koreensis* IB-4 and *P. laurentiana* ANT 17 increased the resistance of plants to the action of the pollutant, stimulated their growth under unfavorable conditions, and also accelerated the process of oil biodegradation. The most significant reduction in the content of hydrocarbons in the soil (by more than two times) was achieved using chickpeas and bacterial strains. However, the natural conditions of the soil environment differ significantly from the model, so in the future we plan to conduct field experiments. Information on the effect of *P. koreensis* IB-4 and *P. laurentiana* ANT 17 strains on the growth and development of leguminous plants, including in oil-containing soil, can be used in the development of biological preparations for agriculture and biotechnologies for eliminating the consequences of hydrocarbon pollution.

## Figures and Tables

**Table 1:** Germination and morphometric indicators of legumes.

Variants of Experiments	Germination (%)	Shoot Length (cm)	Length of the Main Root (cm)	Number of Leaves (pcs)	Number of Nodules (pcs Plant^−1^)	Shoot Length (cm)	Length of the Main Root (cm)	Number of Leaves (pcs)	Number of Nodules (pcs Plant^−1^)
		21 Days				42 Days			
		Peas							
Control	93.3 ± 4.6 ^a^	32.3 ± 1.8 ^a^	11.0 ± 0.3 ^ab^	6.3 ± 0.2 ^a^	2.1 ± 0.1 ^a^	73.7 ± 2.9 ^a^	16.2 ± 0.9 ^a^	12.7 ± 0.5 ^a^	9.0 ± 0.3 ^a^
IB-4	96.7 ± 3.7 ^ab^	38.0 ± 1.0 ^b^	10.8 ± 0.3 ^a^	6.7 ± 0.3 ^a^	1.8 ± 0.1 ^a^	85.5 ± 4.3 ^b^	18.2 ± 1.2 ^a^	15.4 ± 0.5 ^b^	20.4 ± 1.2 ^b^
ANT 17	96.7 ± 3.7 ^ab^	36.8 ± 0.9 ^b^	12.4 ± 0.2 ^b^	5.9 ± 0.1 ^a^	12.0 ± 0.4 ^b^	78.7 ± 3.5 ^ab^	17.9 ± 1.0 ^a^	15.0 ± 0.6 ^b^	20.8 ± 1.2 ^b^
		Lupine							
Control	90.0 ± 4.9 ^a^	14.7 ± 0.6 ^a^	8.3 ± 0.3 ^a^	4.0 ± 0.1 ^a^	0.3 ± 0.1 ^a^	29.1 ± 1.3 ^a^	14.5 ± 0.8 ^a^	10.5 ± 0.4 ^a^	10.2 ± 0.4 ^a^
IB-4	93.3 ± 4.6 ^a^	18.5 ± 0.8 ^b^	8.8 ± 0.2 ^ab^	5.0 ± 0.1 ^a^	- *	34.3 ± 1.5 ^a^	14.6 ± 0.7 ^a^	11.7 ± 0.5 ^ab^	8.3 ± 0.4 ^a^
ANT 17	90.0 ± 4.9 ^a^	18.8 ± 0.7 ^b^	10.2 ± 0.4 ^b^	5.0 ± 0.2 ^a^	0.5 ± 0.2 ^a^	35.3 ± 1.7 ^a^	18.9 ± 1.4 ^b^	12.8 ± 0.5 ^b^	16.1 ± 0.6 ^b^
		Chickpea							
Control	86.7 ± 4.6 ^a^	28.5 ± 1.1 ^ab^	12.7 ± 0.5 ^a^	9.4 ± 0.4 ^a^	-	38.1 ± 2.1 ^a^	17.0 ± 1.1 ^a^	16.0 ± 0.7 ^ab^	-
IB-4	90.0 ± 4.9 ^a^	27.0 ± 0.9 ^a^	15.3 ± 0.7 ^b^	9.7 ± 0.3 ^a^	-	44.0 ± 3.2 ^a^	22.5 ± 1.2 ^b^	14.7 ± 0.6 ^a^	-
ANT 17	90.0 ± 4.9 ^a^	31.1 ± 1.3 ^b^	14.0 ± 0.5 ^ab^	10.6 ± 0.5 ^a^	-	45.2 ± 2.1 ^a^	22.7 ± 1.0 ^b^	17.4 ± 0.8 ^b^	-
		Alfalfa							
Control	71.7 ± 1.8 ^a^	9.3 ± 0.3 ^a^	5.2 ± 0.1 ^a^	3.1 ± 0.1 ^a^	1.9 ± 0.1 ^a^	18.9 ± 0.9 ^a^	15.0 ± 0.6 ^a^	7.2 ± 0.2 ^a^	7.2 ± 0.2 ^a^
IB-4	86.7 ± 2.3 ^b^	9.5 ± 0.5 ^a^	5.9 ± 0.2 ^a^	3.3 ± 0.1 ^a^	3.8 ± 0.2 ^b^	17.8 ± 0.8 ^a^	14.2 ± 0.6 ^a^	8.1 ± 0.3 ^a^	11.1 ± 0.6 ^b^
ANT 17	81.7 ± 1.8 ^b^	8.5 ± 0.3 ^a^	4.8 ± 0.1 ^a^	3.0 ± 0.1 ^a^	3.8 ± 0.2 ^b^	18.4 ± 0.9 ^a^	14.5 ± 0.5 ^a^	8.1 ± 0.2 ^a^	10.4 ± 0.6 ^b^
		Sweet clover							
Control	83.3 ± 2.3 ^a^	7.2 ± 0.2 ^ab^	5.0 ± 0.2 ^a^	2.9 ± 0.1 ^a^	1.5 ± 0.1 ^a^	14.0 ± 0.6 ^a^	14.3 ± 0.7 ^ab^	5.6 ± 0.2 ^a^	12.5 ± 0.6 ^a^
IB-4	90.0 ± 2.8 ^b^	8.3 ± 0.2 ^b^	5.6 ± 0.2 ^a^	2.7 ± 0.2 ^a^	3.0 ± 0.1 ^b^	14.0 ± 0.8 ^a^	12.3 ± 0.5 ^a^	6.0 ± 0.3 ^a^	13.6 ± 0.6 ^a^
ANT 17	86.7 ± 3.7 ^ab^	6.8 ± 0.3 ^a^	4.9 ± 0.2 ^a^	3.4 ± 0.2 ^a^	2.3 ± 0.1 ^ab^	16.5 ± 0.8 ^a^	15.0 ± 0.7 ^b^	7.2 ± 0.3 ^a^	16.6 ± 0.8 ^b^

Statistically different means values for each indicator (n = 10) are marked with different letters (*p* ≤ 0.05). IB-4 and ANT 17 are variants of the experiment with the introduction of *P. koreensis* IB–4 and *P. laurentiana* ANT 17, respectively. * There are no nodules.

**Table 2:** The number of microorganisms in the rhizosphere of leguminous plants, CFU/g.

Variants of Experiments	Peas		Lupine		Chickpea		Alfalfa		Sweet Clover	
	21 Days	42 Days	21 Days	42 Days	21 Days	42 Days	21 Days	42 Days	21 Days	42 Days
	Heterotrophic microorganisms, ×10^7^									
Control	(5.4 ± 0.3) ^a^	(4.4 ± 0.2) ^b^	(3.5 ± 0.2) ^a^	(5.8 ± 0.3) ^b^	(5.3 ± 0.3) ^b^	(3.9 ± 0.2) ^c^	(2.7 ± 0.1) ^b^	(0.9 ± 0.1) ^a^	(1.0 ± 0.2) ^a^	(1.9 ± 0.1) ^a^
IB-4	(4.3 ± 0.2) ^a^	(1.7 ± 0.1) ^a^	(4.3 ± 0.2) ^a^	(3.1 ± 1.2) ^ab^	(3.3 ± 0.2) ^a^	(1.3 ± 0.1) ^b^	(0.6 ± 0.1) ^a^	(2.0 ± 0.1) ^a^	(0.5 ± 0.1) ^a^	(3.1 ± 0.2) ^a^
ANT 17	(5.4 ± 0.2) ^a^	(6.4 ± 0.3) ^c^	(3.2 ± 0.1) ^a^	(1.9 ± 0.1) ^a^	(4.2 ± 0.2) ^ab^	(0.7 ± 0.1) ^a^	(0.7 ± 0.1) ^a^	(2.0 ± 0.1) ^a^	(1.0 ± 0.1) ^a^	(2.9 ± 0.2) ^a^
	Oligonitrophilic microorganisms, ×10^7^									
Control	(1.2 ± 0.1) ^a^	(2.0 ± 0.1) ^a^	(1.6 ± 0.1) ^a^	(1.2 ± 0.1) ^a^	(2.8 ± 0.1) ^a^	(1.1 ± 0.1) ^a^	(1.2 ± 0.1) ^a^	(1.7 ± 0.1) ^a^	(0.8 ± 0.1) ^a^	(1.3 ± 0.1) ^a^
IB-4	(1.6 ± 0.1) ^a^	(1.5 ± 0.1) ^a^	(2.0 ± 0.1) ^a^	(0.8 ± 0.1) ^a^	(3.0 ± 0.2) ^a^	(1.2 ± 0.1) ^a^	(0.5 ± 0.1) ^a^	(1.7 ± 0.1) ^a^	(0.9 ± 0.2) ^a^	(2.5 ± 0.2) ^a^
ANT 17	(1.1 ± 0.1) ^a^	(1.5 ± 0.1) ^a^	(1.7 ± 0.1) ^a^	(2.1 ± 0.1) ^a^	(2.6 ± 0.1) ^a^	(0.8 ± 0.1) ^a^	(0.9 ± 0.1) ^a^	(1.8 ± 0.1) ^a^	(0.8 ± 0.1) ^a^	(1.5 ± 0.1) ^a^
	Micromycetes, ×10^4^									
Control	(8.6 ± 0.5) ^c^	(4.2 ± 0.1) ^b^	(35.0 ± 1.8) ^b^	(3.4 ± 0.2) ^b^	(13.3 ± 0.7) ^c^	(1.4 ± 0.1) ^a^	(11.3 ± 0.7) ^c^	(2.5 ± 0.1) ^a^	(9.8 ± 0.5) ^c^	(2.9 ± 0.2) ^ab^
IB-4	(3.2 ± 0.1) ^a^	(2.5 ± 0.1) ^a^	(2.0 ± 0.1) ^a^	(2.6 ± 0.1) ^ab^	(2.1 ± 0.1) ^a^	(1.8 ± 0.1) ^a^	(0.8 ± 0.1) ^a^	(2.3 ± 0.1) ^a^	(0.5 ± 0.1) ^a^	(3.9 ± 0.2) ^b^
ANT 17	(4.7 ± 0.2) ^b^	(5.0 ± 0.3) ^b^	(2.8 ± 0.1) ^a^	(1.1 ± 0.1) ^a^	(4.9 ± 0.3) ^b^	(1.4 ± 0.1) ^a^	(2.3 ± 0.2) ^b^	(3.6 ± 0.2) ^a^	(1.6 ± 0.1) ^b^	(2.2 ± 0.1) ^a^

Statistically different means values for each indicator are marked with different letters (*p* ≤ 0.05). IB-4 and ANT 17 are variants of the experiment with the introduction of *P. koreensis* IB–4 and *P. laurentiana* ANT 17, respectively.

**Table 3:** The influence of oil pollution on the morphometric parameters of chickpea and alfalfa changeable.

Variants of Experiments	Dry Mass of Shoots (mg)	Dry Mass of Roots (mg)	Root Mass/ Shoot Mass	Number of Nodules (pcs Plant^−1^)
	Chickpea			
Control	54.3 ± 2.3 ^a^	28.5 ± 2.1 ^a^	0.52 ^a^	-*
Oil	56.6 ± 1.8 ^a^	33.3 ± 3.3 ^ab^	0.59 ^ab^	-
Oil + IB-4	66.8 ± 2.5 ^b^	33.7 ± 4.5 ^ab^	0.50 ^a^	-
Oil + ANT 17	62.3 ± 2.6 ^b^	38.6 ± 2.6 ^b^	0.62 ^b^	-
	Alfalfa			
Control	9.75 ± 0.86 ^c^	1.18 ± 0.21 ^a^	0.12 ^a^	6.7 ± 0.2 ^b^
Oil	2.41 ± 0.19 ^a^	1.79 ± 0.14 ^a^	0.74 ^c^	2.8 ± 0.2 ^a^
Oil + IB-4	4.09 ± 0.29 ^b^	2.24 ± 0.19 ^a^	0.55 ^b^	3.7 ± 0.4 ^a^
Oil + ANT 17	3.56 ± 0.47 ^ab^	1.80 ± 0.06 ^a^	0.51 ^b^	3.7 ± 0.4 ^a^

Statistically different means values for each indicator (n = 10) are marked with different letters (*p* ≤ 0.05). IB-4 and ANT 17 are variants of the experiment with the introduction of *P. koreensis* IB–4 and *P. laurentiana* ANT 17, respectively. * There are no nodules.

**Table 4:** The influence of oil pollution on the population microorganisms in the rhizosphere of chickpea and alfalfa changeable, CFU/g.

Variants of Experiments	Heterotrophic Microorganisms, ×10^7^	Oligonitrophilic Microorganisms, ×10^6^	Micromycetes, ×10^4^	Petroleum-Degrading Microorganisms, ×10^5^
	Chickpea			
Control	(3.4 ± 0.7) ^a^	(8.0 ± 0.6) ^b^	(5.3 ± 0.8) ^bc^	(4.4 ± 0.9) ^a^
Oil	(8.5 ± 0.7) ^b^	(0.8 ± 0.3) ^a^	(1.6 ± 0.5) ^a^	(18.9 ± 2.3) ^b^
Oil + IB-4	(15.2 ± 0.9) ^c^	(1.3 ± 0.4) ^a^	(4.5 ± 0.8) ^ab^	(16.3 ± 1.9) ^b^
Oil + ANT 17	(20.4 ± 1.5) ^d^	(1.8 ± 0.4) ^a^	(7.7 ± 0.8) ^c^	(20.8 ± 1.6) ^b^
	Alfalfa			
Control	(1.1 ± 0.1) ^a^	(3.7 ± 0.3) ^b^	(3.1 ± 0.2) ^b^	(1.3 ± 0.1) ^a^
Oil	(2.4 ± 0.1) ^ab^	(0.4 ± 0.1) ^a^	(1.1 ± 0.1) ^a^	(5.0 ± 0.4) ^b^
Oil + IB-4	(6.5 ± 0.3) ^c^	(0.7 ± 0.1) ^a^	(3.5 ± 0.3) ^b^	(5.3 ± 0.3) ^b^
Oil + ANT 17	(4.6 ± 0.3) ^b^	(0.9 ± 0.1) ^a^	(3.5 ± 0.2) ^b^	(4.9 ± 0.3) ^b^

Statistically different means values for each indicator are marked with different letters (*p* ≤ 0.05). IB-4 and ANT 17 are variants of the experiment with the introduction of *P. koreensis* IB–4 and *P. laurentiana* ANT 17, respectively.

## Data Availability

The data presented in this study are available in the graphs and tables provided in the manuscript.
